# Ophthalmology

**Published:** 1891-06

**Authors:** Alvin A. Hubbell


### OPHTHALMOLOGY.

CONDUCTED BY

ALVIN A. HUBBELL, M. D.

### CENTRAL SCOTOMA.

Jensen, of Copenhagen, has published an important work on this subject, an abstract of which he furnished to the Danish *Nordisk Ophthalmologisk Tidsskrift*, Vol. III., No. 1. His investigations were upon cases which had presented themselves at Prof. Hensen Grut's clinic from Oct. 1, 1875, to July 31, 1889. Out of 60,125 patients, 269 were cases of central scotoma. This number included all cases, whether the ophthalmoscopic examination was negative or positive, whether the periphery of the field of vision was normal or not, and whether the affection had arisen suddenly or gradually and in one or both eyes. Cases of amblyopia without restriction of the field of vision, whether absolute or relative defect of the central vision presented itself or not, were not included.

He arranged the 269 cases into the following groups : 1, amblyopia centralis ; 2, stationary scotomatous optic atrophy ; 3, progressive scotomatous optic atrophy ; 4, bilateral optic neuritis with central scotoma ; 5, unilateral amblyopia, neuritis, or atrophy, with central scotoma ; 6, glaucoma simplex.

*Amblyopia centralis* included the majority of all the cases, viz., 189, in three of which it was unilateral. It was characterized by an absence of corresponding changes in the fundus of the eye, by the defect being confined to the central portion of the field of vision where it occurred as a negative scotoma of peculiar shape and extent, which was most readily demonstrable by colors, and by never leading to blindness and often to recovery. It occurred in persons of middle or advanced life for the most part, and the use of tobacco and alcohol was a prominent cause. The scotoma was in no case absolute for a white object $\frac{1}{4}$ to 1 cm. square at a distance of 25 centimeters. The vision varied from $\frac{5}{18}$ to $\frac{5}{60}$. In a few cases it was reduced to counting fingers at 4 or 5 meters. Pathological pallor of the temporal portion of the disc was noticed in seventeen cases, but complete atrophy never occurred.

*Stationary scotomatous optic atrophy* was rare, being represented by twenty cases. It occurred exclusively in young men under the age of thirty-four, usually between the twentieth and twenty-fifth year. It occasionally showed a hereditary tendency, was sometimes apparently caused by want of sleep or other weakening influences, and often came without any demonstrable cause. It occurred suddenly or reached its maximum in a short time, the central amblyopia was considerable and, as a rule, remained unchanged, neither improving nor getting worse, and it developed usually in both eyes simultaneously. Before the lapse of a year, sometimes much earlier, the ophthalmoscope showed complete atrophy of the optic disc. A decided optic neuritis was never found to precede the atrophy.

*Progressive scotomatous optic atrophy* was also rare, being found in only twenty-three cases. Like the stationary variety, this condition was present only in men, but in those who were older, between the fortieth and fiftieth year. The cases were frequently syphilitic, and the premonitory symptoms of tabes dorsalis—absence of tendon reflex, myosis, Argyll-Robertson pupil, etc.,—were frequently seen. The central amblyopia or scotoma came on gradually, first in one eye and then the other. Complete atrophy of the papillæ came on with more certainty and earlier than in the preceding form. The disease was progressive in character and the prognosis bad.

*Bilateral scotomatous optic neuritis* occurred in eleven cases, and differed from the other forms of central scotoma, both as to age and sex of the patients, as well as to symptoms and course.

There was a picture of optic neuritis from intra-cranial affection, and the course was the usual one of this disease. The scotoma was not characteristic of optic neuritis, but was looked upon as the expression of a particular localization of the morbid process in the optic nerve.

*Unilateral central scotoma* was found in twenty-two cases of varying conditions, such as unilateral optic neuritis, hysteria, disorders of menstruation, incomplete embolism of the central retinal artery, etc.

*Glaucoma simplex* furnished two cases.

Jensen believes that the answer to the question as to whether or not central scotoma is always produced by a morbid process in the "papilla-macular" fibers of the optic nerve cannot yet be satisfactorily answered for lack of sufficient post-mortem investigations in this direction. However this may be, the simultaneous occurrence and similar development in both eyes are especially suggestive of a common cause in a higher center.—*Ophthalmic Review*, Jan., 1891.

---

### TREATMENT OF BLEPHAROSPASM.

Allport, of Minneapolis (*Am. Jour. Ophthalmology*, Jan., 1891), has adopted the following method of treating the most intractable and annoying condition known as blepharospasm: Place a strong, short eye-speculum between the lids and open its blades until it is deemed that the muscle is put thoroughly upon the stretch. The speculum is then firmly set and allowed to remain for about five minutes. The same result may be obtained by two lid elevators. As the procedure is quite painful, as a rule, it is better to place the patient under a general anesthetic beforehand.

---

### DIVISION OF ANTERIOR SYNECHIA.

Lang (*London Ophthalmic Hospital Reports*, Dec., 1889, and Dec., 1890,) recommends the division of adhesions of the iris to the cornea by means of a pair of knives—one an ordinary Knapp's discision-knife, the other a similar knife with a blunt point. The eye being cocainized, the lids separated by a speculum, and the globe fixed close to the adhesion, the sharp-pointed knife is passed through the cornea, at a point as far removed as possible from the synechia, in such a manner that the aqueus will not escape on its removal. The blunt-pointed knife is then inserted through the opening thus made in the cornea, and the synechia divided by a gentle sawing motion. The employment of eserine, both before and after the operation, is advised, except where the adhesion is central, when atropia is preferable. Sometimes one or more repetitions of the operation are necessary.

---

### ABSENCE OF THE PATELLAR TENDON-REFLEX IN INTERSTITIAL KERATITIS.

Lang and Wood (*Royal London Ophthalmic Hos. Reports*, Vol. XII., p. 312,) have recorded the results of their examination of sixty-two cases of interstitial keratitis in reference to the presence or absence of the patellar tendon-reflex, from which they conclude:

1. That in about 30 per cent. of all cases of interstitial keratitis the knee-jerk is decidedly subnormal.
2. That in about 10 per cent. of all cases it is entirely absent—all known cases of subnormal tendon-reflexes, outside of the constant fact that the local eye-disease exists, having been eliminated.
3. It is probable also that in a very small percentage of cases of diminished and absent knee-jerks the usual constitutional dyscrasia cannot be demonstrated.
4. That it is rare to find a case of exaggerated patellar tendon-reflex in interstitial keratitis when unaccompanied by some of the affections known to produce the former.

---

### MUSCULAR ASTHENOPIA.

Roosa (*Ophthalmic Review*, October, 1890,) does not believe that muscular asthenopia, as generally understood, has any such importance as writers have attributed to it. Myopia, hypermetropia, and astigmatism are the causes of muscular insufficiencies. An organic fixed condition of the eyeball is the chief cause of asthenopia, and unstable secondary muscular conditions do not deserve much consideration. No fixed standard can be devised by which to measure the power of the muscles of the eye, any more than for other muscles of the body.

